# Relationship between Gingival Inflammation and Pregnancy

**DOI:** 10.1155/2015/623427

**Published:** 2015-03-22

**Authors:** Min Wu, Shao-Wu Chen, Shao-Yun Jiang

**Affiliations:** ^1^Department of Stomatology, The Affiliated Shenzhen Maternity and Child Healthcare Hospital of the South Medical University, Shenzhen 518048, China; ^2^School of Dentistry, Tianjin Medical University, Tianjin 300070, China

## Abstract

An increase in the prevalence and severity of gingival inflammation during pregnancy has been reported since the 1960s. Though the etiology is not fully known, it is believed that increasing plasma sex steroid hormone levels during pregnancy have a dramatic effect on the periodontium. Current works of research have shown that estrogen and progesterone increasing during pregnancy are supposed to be responsible for gingivitis progression. This review is focused not only on epidemiological studies, but also on the effects of progesterone and estrogen on the change of subgingival microbiota and immunologic physiological mediators in periodontal tissue (gingiva and periodontal ligament), which provides current information about the effects of pregnancy on gingival inflammation.

## 1. Introduction

Periodontal health in pregnant women has become a field of research since the 1960s, resulting in a flurry of studies to focus on it [1]. Gingival inflammation associated with pregnancy has been initiated by dental plaque and exacerbated by endogenous steroid hormones [2]. Meanwhile, the bidirectional interaction between systemic conditions and periodontal status has been taken more seriously into consideration with the proposition of periodontal medicine since the middle 1990s [3]. Although it is mandatory to exclude the effects of previously existing periodontal inflammation and dental plaque in order to explore the sole effect of pregnancy on periodontal health, the works of research in this regard have rarely been performed. This narrative review summarizes the current status of epidemiological and mechanistic studies on the changes of periodontium during pregnancy, especially the normal periodontium in order to elucidate the effect of pregnancy on the progress of gingival inflammation.

## 2. Epidemiological Studies

### 2.1. Prevalence

An increase in the prevalence and severity of gingival inflammation during pregnancy without plaque association has been reported since the early 1960s [1, 4, 5]. Clinically, preexisting gingivitis or periodontitis in pregnant women would be worsening dramatically. The periodontal changes are characterized by increasing periodontal probing depths, bleeding upon probing or mechanical stimulation, and gingival crevicular fluid flow, which disappears postpartum [6]. In previous studies, it appears that gingival inflammation shows prevalence from 30% to 100% when pregnancy occurs [7]. Meanwhile, some cross-sectional research showed that the percentage of pregnant women with gingivitis was 89% in Ghana, 86.2% in Thailand, and 47% in Brazil [8–10]. This variation may reflect the different populations studied and their characteristics, as well as the differences in definitions of periodontal disease between studies [8].

### 2.2. Periodontal Changes during Pregnancy

In accordance with previous studies [1, 4–7], recent cross-sectional and longitudinal studies have further confirmed and extended the association between pregnancy and gingival condition in many cultural and ethnic groups. In 2000, a group of researchers reported the findings of the study including 47 pregnant women and 47 nonpregnant women who served as matched controls in a rural population of Sri Lankans [11]. The periodontal status of the pregnant women was evaluated in the first, second, and third trimester of pregnancy and the final examination was at three months postpartum. The authors found that although the plaque levels remained unchanged, the gingival index (GI) of pregnant women was significantly increased and peaked in the third trimester but dropped at 3 months postpartum [11]. The results were consistent with the findings of another cohort study in 2003 consisting of 200 pregnant women and 200 nonpregnant controls in Jordan [12]. In this study, it was reported that pregnant women had significantly higher GI and periodontal pocket depth (PPD) with similar plaque index (PI) compared with nonpregnant women. The clinical parameters (PPD and GI) increased in parallel with the increase in the stage of pregnancy, which reached the maximum at the eighth month [12]. In another companion study with a smaller sample size of 19 pregnant women, bleeding on probing (BOP) decreased from 41.2% at the twelfth week of pregnancy to 26.6% postpartum without any active periodontal therapy [13].

In addition to periodontal clinical parameters as above, clinical attachment level (CAL) measurements were also detected in the recent studies mentioned above. From these studies, the increased inflammation was detected in the gingival region rather than in other periodontal sites, indicating that pregnancy only has reversible effect on the gingiva without inducing periodontal attachment loss. It could be speculated that periodontal attachment loss requires a chronic inflammatory state of the gingiva lasting longer than pregnancy when the gingival changes occur [14]. However, this speculation remains to be proved. The recent studies observing the periodontal condition of women taking combined oral contraceptives (progesterone and estradiol) for at least 1 year have not reached an agreed conclusion about the change of CAL [15–17]. Some results showed that attachment loss was significantly greater in the users of combined oral contraceptives (COC) compared to the nonusers [15, 18]. The others found no difference in CAL between women taking COC and controls [16, 17]. One of the possible explanations for the discrepancy was that these study designs were partly different [19]. More experiments with oral contraceptives and long-term studies are necessary for answering this issue.

Recent studies further confirmed that gingivitis associated with pregnancy seemed to be dependent on, but unrelated to, the amount of dental plaque accumulation [20]. It seemed that good oral hygiene in pregnancy was able to partially neutralize hormonal effect [21]. Although, as it is well known, periodontal diseases have been considered to be microorganisms initiated, whether pregnancy's influence on gingival tissue might be independent or pregnancy by itself would cause new gingivitis has been proposed. Two most recent cohort studies were performed according to this proposal. Differed from those studies described above, these studies included the healthy periodontium without any gingival inflammation and excellent oral hygiene marked with fairly low plaque index in the subject criteria. One of these studies followed 48 pregnant Spanish women with healthy periodontium and examined their periodontal index in the first, second, and third trimesters and at 3 months postpartum. Despite maintaining fairly low Pl values, the pregnant women showed an increase in GI which maintained high levels in the third trimester and then decreased at 3 months postpartum [22]. In the other longitudinal study, the authors described the development of gingival inflammation in 30 periodontally healthy pregnant women with good oral hygiene in Finland. They found that the increase in gingival inflammation evaluated by BOP and the number of deep periodontal pockets (PPD ⩾ 4 mm) in pregnant women was not related to dental plaque simultaneously between the first and second trimesters, followed by a decrease afterwards [23]. These two studies tried to wipe off the effects of previously existing gingival inflammation and dental plaque accumulation on the progress of pregnancy gingival inflammation. From these two studies, the increase in inflammatory changes of gingiva was mainly induced by pregnancy. The results further confirmed the possibly negative influence of pregnancy on periodontal situation. However, it is clear that it is difficult to keep the teeth without any plaque. Thus, the most persuading and powerful study should be based on plaque-free experimental animal models.

No matter whether the plaque levels remained unchanged or low, the concept that a progressive increase in gingival inflammation without periodontal attachment loss during pregnancy and apparent decrease following parturition is strengthened by these data from most studies. However, there are still a few works of research denying the association between pregnancy and gingival inflammation. Miyazaki et al. observed that there was no difference in periodontal status between pregnant and nonpregnant women in a study using the CPITN index to assess the periodontal conditions of 2424 pregnant and 1565 nonpregnant women. In addition, to observe that 95% of the pregnant women and 96% of the nonpregnant women had some signs of periodontal disease, the authors also noticed that pregnant women even had a healthier periodontal condition; that is, the number of sextants with healthy periodontal tissues was higher, the percentage of people having deep pockets (6 mm or deeper) was lower, and the need for prophylaxis was lower in pregnant than in nonpregnant women [24]. The difference of populations, the criteria for defining healthy periodontal condition, the clinical measurements used, and the numbers of teeth examined may complicate the results of these observations. Similarly, Jonsson and his colleagues found that none of the periodontal parameters for the pregnant females differed significantly from those of nonpregnant females. These parameters showed no significant correlation with the progression of pregnancy [25]. Since the findings were based on a small sample size of 9–14 subjects, there is the limitation in this study.

## 3. Mechanistic Studies

### 3.1. Estrogen, Progesterone, and Their Receptors

The exact mechanisms for the onset of the greater gingival inflammation during pregnancy have not yet been clearly described. Since the 1970s, the obvious increase in circulating levels of estrogen and progesterone was considered to have a dramatic effect on the periodontium throughout pregnancy and be correlated with this clinical feature [26]. The principal estrogen in plasma is estradiol, which is produced by the ovary and the placenta. The principal progestin in female is progesterone, secreted by the corpus luteum, placenta, and the adrenal cortex [7]. During pregnancy, both of them are elevated due to continuous production by the corpus luteum at the beginning and the placenta afterward. By the end of the third trimester, progesterone and estrogen reach the peak plasma levels of 100 and 6 ng/mL, respectively, which are 10 and 30 times the levels observed during the menstrual cycle [27]. In animal models, the physiological effect of estrogen on gingiva was also observed [28]. When the serum estrogen concentrations in baboons were suppressed below 100 pg/mL by the administration of aromatase inhibitor, gingival enlargement developed. The gingiva recovered clinically when estradiol was added. The results indicated that estrogen profoundly affects physiologic events in the gingiva, including cellular proliferation and differentiation, whether directly or indirectly. Another report showed that the estrogen level determined the level of gingival margin inflammation developing against microbial plaque [29], when detecting 30 pregnant and 24 nonpregnant females. From above, both too low and too high estrogen levels have harmful effect on the gingiva.

Studies that investigated the impact of sex steroids on the periodontium are supported by the following observations. Localization of estrogen receptor (ER) and progesterone receptor (PgR) has been reported in the human periodontium, demonstrating that the periodontal tissues are the target tissues for these hormones [30]. Also, in earlier reports, ER was found in the periodontium of human, including gingival and periodontal ligament [30, 31]. However, using polymerase chain reaction analysis, Parkar et al. did not detect the expression of ER in any of the periodontal or gingival tissue samples [32]. The discrepancy was explained by the authors with the lack of specificity of the techniques used in previous experiments. In addition, the receptor subtypes were not specially examined in earlier reports [7].

Recent studies have further demonstrated the localization and subtypes of estrogen and progesterone in periodontium. Kawahara and Shimazu have reported that human GFs expressed poor ER-*α* signal but chiefly expressed ER-*β*. This was speculated to be the first description of the ER subtype in gingival component cells by the authors [33]. Jönsson and colleagues in their serial studies confirmed the ER subtypes in periodontal tissue [34]. ER-*β* immunoreactivity was observed in the nuclei of about 40% of cultured human PDLCs, while no ER-*α* immunoreactivity was detected, suggesting that estrogen influences the functional properties of periodontal ligament cells preferentially through ER-*β*. According to the authors, this was the first report revealing that ER-*β* is expressed in human PDLCs [34]. Recently, it was further suggested that ER-*β* localize not only in nuclei but also in mitochondria of human PDLCs, demonstrating that estrogen, probably via ER-*β*, influences mitochondrial function and energy metabolism in human PDLCs [35]. In addition, Välimaa et al. reported that gingival epithelial cells in healthy gingiva expressed the ER-*β* protein [36]. Nebel et al. further found that ER-*β* was located not only in nuclei of epithelial cells in all layers of the gingival epithelium, but also in cells of the lamina propria [37]. It could be concluded that ER-*β* was the predominant ER in periodontium, implying that the effects of estrogen on gingival tissues were mediated by ER-*β* [37].

However, the discrepancy exists in the expression of PgR. Jönsson et al. found that no PgR was expressed in human PDLCs [38]. Kawahara and Shimazu reported that human GFs expressed low PgR expression [33]. In a recent study in China, the authors detected the expression of PgR in human PDLCs by reverse transcriptase-polymerase chain reaction and immunocytochemistry, which showed that the PgR was expressed in human PDLCs at the gene and protein levels [39]. The staining methods and procedures, cell source, age of donors, and menstrual cycle stage might explain the discrepancies between the results. Taken collectively, it is clear that the periodontium is a target tissue for estrogen and progesterone, although the presence of PgR has not been conclusively demonstrated in these tissues.

Periodontium is a unique structure composed of two fibrous (gingival and periodontal ligament) and two mineralized (cementum and alveolar bone) tissues [7]. For the reason that pregnancy probably has an effect only on the gingiva and has no permanent effects on periodontal attachment, meantime, the effect of female sex hormones on periodontal ligament and tooth supporting alveolar bone has rarely been investigated [40]; this paper mainly focuses on the impact of progesterone and estrogen on two fibrous tissues (gingival and periodontal ligament) and a review of the impact of hormones on alveolar bone is not given here.

### 3.2. Alterations in Subgingival Microbiota

It is widely agreed that the majority of tissue damage in gingivitis and initial periodontal lesions occurs via an inflammatory response of the host to the presence of microbes, their structural and metabolic products, and the products of affected tissues themselves [41]. Pregnancy-associated gingivitis is no exception. It has been suggested that estrogen and progesterone can modulate the putative periodontal pathogens, the immune system in the gingiva, the specific cells in the periodontium, and the gingival vasculature [7, 8]. Recent studies were mainly performed to investigate the influence of pregnancy on microbial organisms and host response factors related to pregnancy gingivitis formation.

Periodontium acts as a reservoir of subgingival bacteria. Changes in the subgingival microbiota have been proposed as a potential mechanism for exacerbated gingival inflammation during pregnancy. In this regard, it should be kept in mind that there are three classic works of research in the early eighties of the last century. In one longitudinal study of 20 pregnant women, Kornman and Loesche were the first to report statistically significant increases in the levels of* Bacteroides intermedius* during the second trimester, with a reduction during the third trimester and after delivery. The marked increase in the proportion of the bacteria seemed to be associated with increased serum levels of progesterone or estrogens which substituted for the naphthaquinone requirement of the pathogens and thus acted as a growth factor for the bacteria [6]. In their following research in vitro, both estradiol and progesterone were involved in the fumarate reductase system of subspecies of* Bacteroides intermedius* and therefore appeared to have potential to alter the subgingival microbial ecology by directly influencing the metabolic pathways of these pathogens [42]. Also, in one cross-sectional study, Jensen et al. reported a 55-fold greater level of* Bacteroides* species during pregnancy over nonpregnancy and 16-fold increase in those taking contraceptives over the control group [43]. Not all the early studies corroborated these findings. As shown in an early assessment, Jonsson et al. found no difference in the levels of* Bacteroides intermedia* between pregnant and nonpregnant controls or any correlation with the progression of the pregnancy [26]. Jonsson's findings led to the speculation that the increase in* Bacteroides intermedia *during the second trimester of pregnancy may actually be independent of estrogens or progesterone and occur for other reasons [8]. Similarly, the small sample size was the limitation of this study.

With the taxonomic evolution of* Bacteroides* species and the development of the molecular method, recent research provided new information on alterations in subgingival microbiota. In the open cohort study, Carrillo-De-Albornoz et al. reported that the worsening gingival inflammation was associated with the presence of subgingival* Porphyromonas gingivalis* and* Prevotella intermedia*, which were positively correlated with maternal hormone levels during pregnancy [44]. However, the proportions of the subgingival periodontal pathogens did not differ throughout pregnancy, although significant differences were found for all the pathogens after delivery [44]. Based on a small sample of pregnant women, Adriaens and coworkers reported the changes in subgingival microflora by DNA-DNA hybridization for 37 species and found that the quantities of* Porphyromonas gingivalis* and* Tannerella forsythia* at the 12th week of pregnancy were associated with gingivitis measured by BOP. No differences in the levels for any of the 37 bacterial species were found between 12th and 28th weeks of pregnancy, although a decrease in 17 of 37 species was found between the 12th week and postpartum, including* Prevotella intermedia* [45].

Many studies mentioned above have employed subgingival bacteria plaque as samples, including those from paper points or curettes. In other recent studies, there is another kind of sample available for measuring the number of oral bacteria, which is saliva sample. According to Umeda et al., whole saliva samples have been reported to contain subgingival periodontopathogens and thus represent an excellent alternative to sampling individual periodontal pockets, which is superior to taking periodontal pocket samples to detect* Porphyromonas gingivalis*,* Prevotella intermedia*,* Prevotella nigrescens*, and* Treponema denticola* in the oral cavity. It might be that whole saliva samples simply contain higher concentrations of the bacteria than a periodontal pocket sample suspended in 0.4 mL water to be detected by PCR [46]. A recent cross-sectional case-control study by Yokoyama et al. used unstimulated saliva of pregnant women to detect periodontopathogens, including* Prevotella intermedia*,* Campylobacter rectus*,* Porphyromonas gingivalis*,* Aggregatibacter actinomycetemcomitans,* and* Fusobacterium nucleatum*. The results showed that* Campylobacter rectus* tended to be higher in pregnant women than in nonpregnant women. The level of* Campylobacter rectus* was positively correlated with the estradiol concentration in the pregnant women [47]. The authors explained the reason of the growth of* Campylobacter rectus* as formate enhancement from the growth of* Prevotella intermedia* that was stimulated by direct interaction of female sex hormones on the fumarate reductase system. Also, another study showed that the growth of* Campylobacter rectus* was significantly enhanced by incorporating either estradiol or progesterone in human gingival fibroblasts (HGF) [48]. However, the authors failed to find that* Prevotella intermedia* were related to signs of gingival inflammation or estradiol concentrations in the saliva, which was not corroborated in other studies [25, 43–45]. This discrepancy could be due to different types of samples (unstimulated saliva compared with subgingival plaque) used and the occurrence rate of* Prevotella intermedia* which seemed to be slightly higher in subgingival sites than in unstimulated saliva [47]. The previous study suggested that the stimulation by masticating a piece of paraffin may increase the outflow of gingival crevicular fluid from the periodontal pocket, which loose the attached microorganisms or clumps of microorganisms from oral biofilms into salivary sediment and then may artificially increase the concentration of components in the saliva [49]. However, there is different opinion about this point. Gürsoy et al. considered that the collected and stimulated saliva contained a higher proportion of glandular saliva, diluting the concentration of gingiva-derived components [50]. This opinion was also proved by their serial longitudinal study [51]. The authors collected subgingival plaque and stimulated saliva samples from periodontally healthy Finnish women and examined them for the presence of* Prevotella intermedia*. In the saliva samples, the proportions of salivary* Prevotella intermedia *did not differ significantly either within the subject group or between the two groups. In subgingival plaque, the level of* Prevotella intermedia* increased transiently twice in the pregnant group, reaching the highest peaks during the second trimester, although the differences were not significant.

It should be noted that the bacteria known as* Fusobacterium nucleatum* were referred to in some aforementioned studies. As an opportunistic oral bacterium, it is associated with various forms of periodontal diseases, including gingivitis. Recently,* Fusobacterium nucleatum* has been gaining increasing attention because of its association with adverse pregnancy outcomes. It is capable of invading not only gingival epithelial cells, gingival fibroblasts, and periodontal ligament fibroblasts, but also other different types of human cells [52, 53]. Unlike other periodontal pathogens, translocation of* Fusobacterium nucleatum* in the acute infection model is organ-specific, that is, only in the placenta, likely due to the immune suppression in the placenta [54]. The recent report of a term stillbirth caused by oral* Fusobacterium nucleatum* provided the first human evidence that the bacteria originated from the mother's subgingival plaque and translocated to the placenta and fetus, causing acute inflammation leading to the fetal demise [55]. Some other authors also focused on the comparison of* Fusobacterium nucleatum* in their works of research. In the two cross-sectional studies aforementioned, no differences were noted in* Fusobacterium* species between pregnant and nonpregnant women [43, 47]. Yokoyama's research further found the correlation between* Fusobacterium nucleatum* and the parameters such as estradiol concentrations and sites (PD = 4 mm), though they found female sex hormones did not promote the growth of* Fusobacterium nucleatum* in their previous in vitro study [47, 48]. Therefore, it was hypothesized that the increased number of PD = 4 mm sites in the pregnant women may have the growth of* Fusobacterium nucleatum*. However, this hypothesis was not consistent with their early findings that both the pregnant and nonpregnant women were comparable in terms of the level of* Fusobacterium nucleatum* [47]. In Adriaens et al.'s longitudinal study, no changes in* Fusobacterium nucleatum naviforme* and* Fusobacterium nucleatum polymorphum* occurred between 12th and 28th weeks of pregnancy. However, both of them decreased greatly at 4 to 6 weeks postpartum. BOP at 12th week is associated with higher counts of* Fusobacterium nucleatum naviforme* and* Fusobacterium nucleatum polymorphum* [45].

Taken together, there is no definite evidence linking increased concentrations of estrogen or progesterone during pregnancy with certain periodontal pathogens. Most works of research focused on* Bacteroides* species have equivocal results, in spite of different methods and different nomenclatures. Studies are needed to further elucidate the change of subgingival microbial profile of pregnant women.

### 3.3. Changes in Host Immunoinflammatory Response

Immunological changes have long been considered to be, at least in part, responsible for periodontal conditions observed during pregnancy [6]. In the various immune mechanisms in the process of gingival inflammation, polymorphonuclear leukocytes (PMNs) are the primary effector cells and appear to play a major role. When stimulated by bacterial pathogens, host cells release proinflammatory cytokines as a part of the immune response. These cytokines recruit PMNs to the site of infection, releasing a variety of biologically active products, such as chemokines, proteolytic enzymes, cytokines, and reactive oxygen species (ROS) [56, 57], and thus indirectly contribute to increase of gingival inflammation. PMNs have been considered to be protective in periodontal disease [58]. It is generally agreed that the damage to periodontal tissue may be aggravated by depressed function of PMNs [59]. During pregnancy, some degree of immunosuppression was reported, which minimizes the risk of fetal rejection [60]. Increased concentrations of female sex hormones may modulate the function and activity of PMNs. Impaired neutrophil functions have been observed throughout pregnancy and are considered to be linked to an increased susceptibility to inflammation [61–64]. Furthermore, human GFs and PDLCs, which are active participants in the oral immune defense system, far from being primarily supporting cells, may potentially produce chemokine signals, proteinases, and cytokines when exposed to suboptimal concentrations of stimuli or to relevant inflammatory cytokines, which associated with periodontal disease [65–68]. Accordingly, the data about the alteration in chemotaxis, cytokines, enzymes, and antioxidant secreted from PMNs, human GFs, or PDLCs in response to the inflammatory stimuli during pregnancy are reviewed in this chapter.

#### 3.3.1. Chemotaxis

In an in vitro study, Miyagi et al. found that progesterone significantly enhanced the chemotaxis of PMNs at a concentration of 200 ng/mL and low concentrations of estradiol reduced it at 0.4 ng/mL which is the most effective concentration, while estradiol and progesterone did not alter chemotaxis of monocytes at any concentration tested [59]. In C. A. Lapp's and D. F. Lapp's recent in vitro study, the chemokines produced by human GFs in response to interleukin-1*β* (IL-1*β*) were significantly inhibited by medroxyprogesterone acetate (MPA) [65]. More recently, Nebel and coworkers investigated the effects of estrogen on the production of chemokines from PDLCs treated with lipopolysaccharide (LPS) and found that a physiological concentration of the endogenous estrogen (100 nm 17*β*-estradiol, which was the same concentration of E2 observed in plasma during pregnancy) differentially regulated chemokine expression in human PDL cells. The results showed that estrogen induced downregulation of chemokine ligand 3 (CCL3) mRNA and upregulation of chemokine ligand 5 (CCL5) gene activity in PDLCs while the expression of chemokine ligand 2 (CCL2) was unaffected by estrogen [68].

#### 3.3.2. Cytokines

The hormonal modulation of effects on cytokines in periodontium has been studied extensively. In Miyagi et al.'s following serial in vitro studies, they concluded that monocytes probably played a role in gingival inflammation more through their release of a variety of cytokines than through their migration to the inflamed lesion. Prostaglandin (PG) E2 by LPS-stimulated human monocytes was enhanced by progesterone at both 2.0 and 20 ng/mL and was reduced by estradiol at 0.4 ng/mL but enhanced at 20 ng/mL. IL-1 was also shown to be inhibited by estradiol and progesterone in a dose-dependent manner [69, 70]. Recently, Yokoyama et al. found that production of interleukin-6 (IL-6) and interleukin-8 (IL-8) by human GFs was enhanced significantly by the stimulation with both estrogen and progesterone at high concentrations comparable to those found in plasma of pregnant women in their study, which suggested that the capacity of female sex hormones to enhance cytokines production by human GFs has the potential to contribute to periodontal disease progression during pregnancy [48]. However, an in vitro study by Lapp et al. has shown that sex hormones had an inhibitory effect on the secretion of IL-6 production by human GFs in response to IL-1 and high levels of progesterone during pregnancy affected the development of localized inflammation by reducing the production of IL-6 [71]. Another in vitro study has also shown that sex hormones at physiological concentrations (E2 of 10^−9^ to 10^−7 ^M) had an inhibitory effect on the secretion of proinflammatory cytokines, including tumor necrosis factor-*α* (TNF-*α*), IL-1*β*, and IL-6 by human PDLCs treated with* E. coli* LPS [72]. Smith et al. also found that TNF-*α* levels in blood neutrophils decreased during the menstrual cycle when estrogen and progesterone concentrations were elevated, supporting a potential anti-inflammatory effect of ovarian hormones on neutrophils [73]. These studies suggested an anti-inflammatory effect of sex hormones at high levels in vitro. However, Jönsson et al. did not find that LPS-induced IL-6 production by human PDLCs was reversed by a physiologically high concentration of E_2_ (100 nM) in human PDLCs, suggesting that estrogen did not exert an anti-inflammatory effect [74]. The in vitro studies mentioned above focused on the effect of sexual hormones on cytokines in periodontal tissue were under the challenge of bacteria. Due to different concentration of ovarian hormones and different experimental protocol, the results were inconsistent.

Despite numerous in vitro studies evaluating the hormonal modulation of effects on cytokines in periodontium, only a few human studies have investigated the change of local proinflammatory mediators in pregnant patients until now [13, 75–77]. In Figuero's cohort study [16], the salivary sexual hormones and gingival crevicular fluid (GCF) levels of a panel of cytokines in samples collected from 48 pregnant women with healthy periodontium were assessed. They found that the levels of IL-1*β* and PGE_2_ showed no significant changes during pregnancy, though their concentrations were higher than those found in nonpregnant women. Exacerbated gingival inflammation during pregnancy could not be associated with changes in PGE2 or IL-1*β*. But, as reported by the authors, the high incidence of dropouts and the lack of homogeneity between the groups might be the limitations of their study [16]. This result corroborated the findings of one cohort study with only 19 pregnant women by Bieri et al., who also found no significant differences in the expression of IL-1*α*, IL-1*β*, IL-8, and TNF-*α* in GCF between week 12 and postpartum, interpreting that the changes in gingival inflammation indicated by BOP may only be weakly associated with the expression of these selected cytokines in GCF during pregnancy [13]. However, the periodontium of the patients in the study was not defined to healthy periodontium before pregnancy as in the former study. Additionally, some cross-sectional studies also found that some proinflammatory mediators may not be associated with gingival inflammation during pregnancy. Otenio et al. found no differences in the expression levels of IL-1*β*, IL-6, and TNF-*α* in pregnant women with and without periodontal disease in comparison with expression of the same genes in nonpregnant women with and without periodontal disease, suggesting that periodontal disease is not influenced by pregnancy [77]. Interestingly, the authors found an apparent reduction in the expression of IL-6 in pregnant women with periodontal disease compared to that in pregnant women without periodontal disease, which is in agreement with the previous in vitro study mentioned above that reported that high levels of progesterone during pregnancy had an inhibitory effect on the secretion of IL-6 by human GFs in response to IL-1 [71].

Similar to the changes of GCF cytokine levels during pregnancy obtained from various works of research, some results were also reported in recent cohort studies evaluating GCF levels of cytokines in the menstrual cycle of periodontally healthy women. In a longitudinal study with 18 periodontally healthy premenopausal women exhibiting stable menstrual cycles, Markou and coworkers found that only IL-6 GCF levels were significantly different between ovulation and progesterone peak, and the subclinical increase of IL-6 at progesterone peak was not accompanied by clinical changes in the periodontium [78]. This result is partly consistent with Becerik et al.'s research among periodontally healthy subjects. The levels of inflammatory markers in GCF were similar in different phases of the menstrual cycle, though the patients had elevated gingival inflammation measured by BOP in ovulation (OV) and menstruation (ME) compared to premenstrual (PM) phases [79]. Inconsistent results existed in Baser et al.'s research, which evaluated the IL-1*β* and TNF-*α* levels in GCF during the menstrual cycle among pregnant women with excellent plaque control. The study showed that IL-1*β* levels in GCF and BOP scores increased significantly from the menstruation day to the predominant progesterone secretion day [80]. These discrepancies can be partially explained by differences in patient selection criteria and time point of clinical sampling [79].

Matrix metalloproteinases (MMPs) are involved in periodontal destruction. However, their role in pregnancy gingivitis is not well studied. In 2010, Gürsoy and coworkers first demonstrated the relationship between the changes of neutrophilic enzymes in saliva and GCF and periodontal status during pregnancy and postpartum in their longitudinal study series [50]. Results showed that a significant reduction of paraffin-stimulated salivary MMPs and tissue inhibitor of matrix metalloproteinase- (TIMP-) 1 expression occurred, despite the increased inflammation and microbial shift towards anaerobes. The increased gingival inflammation was not reflected by the enzymes examined in GCF. MMP-8 and PMN elastase levels of GCF stayed steadily at low levels during pregnancy, despite increasing BOP and PD scores. Their results are supported by some in vitro studies. Lapp et al. showed that progesterone may control and reduce local production of MMPs by cultured human GFs in response to interleukin-1 [81]. Smith et al. also found that MMP-9 levels in blood neutrophils decreased during the menstrual cycle when estrogen and progesterone concentrations were elevated [73]. The reduction of proteinase concentrations in local tissues, including saliva and GCF, may show impairment of neutrophil functions during pregnancy, which may partially explain induced or enhanced susceptibility to gingivitis during pregnancy. In addition, these findings could explain, at least in part, the reason that pregnancy gingivitis itself does not predispose or proceed to periodontitis.

#### 3.3.3. Oxidative Stress

Oxidative stress is a mediator through which immune response in periodontium and pregnancy may be linked. Pregnancy is inherently a state of oxidative stress arising from the increased metabolic activity in placental mitochondria and production of reactive oxygen species (ROS), mainly that of superoxide anion (O^2−^). Meanwhile, scavenging power of antioxidants is reduced [82]. Oxidative stress also plays a significant role in the pathology of periodontal diseases [83]. Imbalance between oxidative stress and antioxidants may play a role in the pathogenesis of periodontitis. Individuals with periodontal disease display high levels of local and systemic biomarkers of oxidative stress [84, 85]. Subjects with worse periodontal health tend to have greater oxidative injury [86]. Recently, the possible relationship among maternal periodontal condition, maternal oxidative stress, and pregnancy has been the subject of several studies. Hickman and colleagues, in a large prospective cohort of healthy pregnant women, examined whether maternal periodontal disease was associated with oxidative stress measured by serum 8-isoprostane. Results indicated that the presence of moderate to severe periodontal disease was significantly associated with increased maternal serum 8-isoprostane, suggesting that maternal periodontal disease was associated with higher oxidative stress during pregnancy [87]. In their earlier report with the same study population, they first reported that periodontal disease and preeclampsia may be linked through maternal systemic oxidative stress measured by serum 8-isoprostane [88]. This may explain their early report in 2008. They found that maternal periodontal disease with systemic inflammation measured by C-reactive protein was associated with an increased risk for preeclampsia [89].

On the other hand, the antioxidant capacity of saliva and gingival crevicular fluid contributes largely to the protection of periodontium against oxidative stress [90]. However, relatively few studies have focused on the change of antioxidant capacity in periodontium during pregnancy. In 2009, Akalin and collaborators, in their longitudinal study, first investigated the periodontal status and antioxidant (AO) defenses during pregnancy. Serum and GCF total AO capacity and superoxide dismutase (SOD) enzyme concentrations were compared among the pregnant patients with chronic periodontitis (CP), pregnant patients with gingivitis (PG), periodontally healthy pregnant women (P-controls), nonpregnant women with CP, and nonpregnant periodontally healthy women. The results showed that systemic and local GCF AO levels decreased in pregnancy and periodontitis, and AO defense reached the lowest level in the last phase of pregnancy, whereas periodontal status deteriorated. The same occurred with SOD. Notably, in periodontally healthy pregnant women, compared to pregnant women with periodontal disease, AO and SOD levels in GCF were higher at the beginning of the pregnancy, but the difference in the third trimester was not statistically significant, suggesting that the GCF AO levels decline in pregnancy was influenced more by pregnancy than by periodontal inflammation, indicating that pregnancy may be a risk factor for the inflammation of periodontium [91]. However, a cross-sectional study performed on a group of pregnant women with or without diabetes has shown some different findings. In this study, Surdacka and colleagues collected unstimulated whole mixed saliva and evaluated the antioxidant system measured by catalase activity. Compared with the healthy individuals, pregnant women with diabetes were found to have markedly increased plaque formation and gingival and periodontal status, as well as increased salivary antioxidant capacity and proinflammatory cytokine levels, which indicated the ongoing inflammatory reaction. These parameters did not seem to correlate with healthy pregnant women. The authors speculated that infection could be taken as a source of oxidative stress that triggered an increase in salivary antioxidant defense [92]. The possible explanation for the disparity between the two studies is the differences in the length of the study period, the mediator measured, and the health status of the study subjects collected. In patients with long-term disease and systemic complications, it is unclear whether oxidative stress is causative for or is a result of these conditions.

Totally, the changes of chemotaxis, cytokines, enzymes, and antioxidants in periodontium during pregnancy are still unclear, regardless whether they are from GF, PDLC, or PMNs. It is speculated that the sexual hormones may exert both anti-inflammatory and proinflammatory effects on the periodontium in a dose-dependent manner. Thus, the gingiva in pregnancy is rendered less efficient at resisting the inflammatory challenges produced by bacteria. At the same time, gingivitis in pregnancy is limited and does not predispose or proceed to periodontitis.

### 3.4. Influences on Cells of the Periodontium

The function of cells in periodontal tissue may be affected by estrogen and progesterone. In an early report, sex steroid hormones have been shown to directly and indirectly exert influence on cellular proliferation, differentiation, and growth in gingiva [6]. In Mariotti's recent study, cellular proliferation and the number of cells entering the S-phase of the cell cycle were significantly increased in the cultures of human premenopausal gingival fibroblasts stimulated by physiologic concentrations of estradiol (1 nM), while both collagen and noncollagen protein productions were reduced [93]. Nebel et al. found that estrogen attenuated proliferation of human gingival epithelial cells monitored by measuring DNA synthesis at high (500 nM and 10 *μ*M) but not low (10 nM) concentrations of estradiol, suggesting a concentration-dependent mechanism [37]. The effects of E2 on hPDL cells were also studied. In recent research by Mamalis, a significant increase in hPDL cell proliferation occurred after estradiol stimulation (100 nM), while cell proliferation did not change after blocking ER-*β* by the short interfering RNA (siRNA) technique. However, collagen synthesis remained unaffected by estradiol stimulation in both stable transfected and nontransfected cells [94]. This observation confirms the results of the previous study that failed to show that estrogen at physiological concentrations (100 nM or lower) mediated significant alterations in collagen synthesis of periodontal ligament cell [38]. However, the physiological concentration (100 nM) of E2 was found to enhance DNA synthesis in human breast cancer MCF-7 cells, suggesting that the effects of estrogen on collagen synthesis are cell/tissue specific [38]. In summary, the data presented here suggest that there is no stimulatory effect of estrogen on the relative amount of collagen synthesized by gingival fibroblasts, PDL cells, or gingival epithelial cells. Also, the stimulatory effects of estrogen on gingival cellular proliferation exist in a concentration-dependent manner.

Due to the uncertainty of location of progesterone receptor in periodontal tissues, the effect of progesterone on cells of the periodontium is far from being determined. There is insufficient information available concerning this regard. Though in low levels, PgR was reported in human GFs, suggesting that progesterone should have an effect on their function [33]. In an in vitro study, an inhibitory effect of progesterone on the proliferation rate of human GFs was observed. Progesterone at concentrations of 50 and 100 *μ*g/mL significantly reduced cellular growth in both cultures derived from a healthy and a diabetic (type II) individual, therefore partly explaining the unfavorable effects of hormonal changes during pregnancy on the gingival tissue [95]. Yuan et al. suggested that progesterone stimulated the proliferation and differentiation of the human PDLCs by PgR [39]. However, Jönsson et al. implied that progesterone does not have a direct effect on PDLCs function; for no nuclear PgR, immunoreactivity was observed in PDLCs [38].

## 4. Conclusion

Based on the data described above, the connection between increased plasma levels of pregnancy hormones and a decline in periodontal health status exists. In addition, the influence of sex hormones can be minimized with good plaque control. From above, it can be assumed that the fluctuation in estrogen and progesterone levels during pregnancy exerts the influence of subgingival microbiota and a spectrum of inflammatory responses in gingival tissues through the changes of chemotaxis, cytokines, enzymes, and antioxidants from PMNs, GFs, and PDLCs and thus indirectly contributes to increased gingival inflammation. The mechanisms responsible for these changes are not fully known. Thus, further works of research are needed to fully elucidate the exact molecular mechanism linking periodontal condition with pregnancy.
